# COVID-19 and sustainable development goals

**DOI:** 10.2471/BLT.20.263533

**Published:** 2020-10-01

**Authors:** Kristin Heggen, Tony J Sandset, Eivind Engebretsen

**Affiliations:** a Centre for Sustainable Healthcare Education, University of Oslo, Klaus Torgaards vei 3, 0372, Oslo, Norway.; b Faculty of Medicine, University of Oslo, Oslo, Norway.

The World Health Organization officially declared the outbreak of the coronavirus disease 2019 (COVID-19) a public health emergency of international concern on 30 January 2020.^1^ A few months later, the world is dealing with a crisis of immense proportions. The pandemic has shown that this crisis is fuelled by poverty, hunger, weak health systems and lack of clean water and sanitation, education and global cooperation.^2^^,^^3^ The global recession caused by the COVID-19 response is alarming and has made researchers question whether the sustainable development goals (SDGs) are fit for the post-pandemic age.^4^ Some have even claimed that certain SDG targets might be counter-productive because they enhance growth rather than development.^4^ While the SDGs do not have a dedicated pandemic response plan, we believe it is essential not to de-link the response to the pandemic from the SDGs. We argue that the COVID-19 crisis demonstrates the need to integrate the SDGs at the national level as well as in individual health-care decisions. We also call for a focus on sustainable health decisions, meaning decisions that are made in the present do not compromise future needs, whether local or global. Making such decisions requires adapting to the current context, anticipating future impact, and using a rights-based framework. 

*Transforming our world: the 2030 agenda for sustainable development*^5^ emphasizes that achieving the SDGs requires balancing three dimensions of sustainable development: economic growth, social inclusion and environmental protection. Moreover, sustainable development requires us to balance our needs with the ability of future generations to meet their own needs. 

Although the SDGs were the outcome of dialogues held at all levels of government and civil society, the related discourse has been criticized for an assumption that governments can and should be the primary custodians of any sustainable development agenda.^6^ This assumption risks consolidating a macro-oriented understanding of sustainable development – that is, that sustainable development concerns only nations, not individuals.

The COVID-19 pandemic shows that sustainable development goes beyond national strategies. Every individual needs to make health decisions that meet personal needs as well as the needs of the broader community, such as using facemasks on public transport, observing social distancing advice and self-quarantining when necessary. Such decisions can help to curb transmission and reduce illness, deaths and economic impacts.

Similarly, this global crisis reveals that community needs can be immediate in contrast to the 2030 horizon of the SDGs. Lack of health insurance, reduced access to water during lockdown situations or chronic diseases have suddenly become factors that determine chances of survival. To manage the COVID-19 pandemic, governments have had to balance the need for mitigation, control and eradication. Should such responses entail enforcing states of emergency, or should they involve a mitigation strategy built on the premise of eventually reaching herd immunity, while being sustainable over time?^8^^,^^9^ How could emergency strategies be consistent with targets such as universal health coverage that might reduce the risk for future pandemics?

Such issues must be addressed in national strategies and in the individual choices that we all make when we comply with health authorities’ recommendations.
